# Different levels of circadian (de)synchrony ­– where does it hurt?

**DOI:** 10.12688/f1000research.127234.1

**Published:** 2022-11-15

**Authors:** Ankita AS. Galinde, Faheem Al-Mughales, Henrik Oster, Isabel Heyde

**Affiliations:** 1 Institute of Neurobiology, Center of Brain, Behavior and Metabolism, University of Lübeck, Lübeck, 23562, Germany; 2 Biochemistry Department, Faculty of Medicine and Health Sciences, Taiz, Yemen

**Keywords:** Chronodisruption, circadian misalignment, coupling, inter-tissue desynchrony, intra-tissue desynchrony, phase incoherence, shiftwork

## Abstract

A network of cellular timers ensures the maintenance of homeostasis by temporal modulation of physiological processes across the day. These so-called *circadian clocks* are synchronized to geophysical time by external time cues (or *zeitgeber*s). In modern societies, natural environmental cycles are disrupted by artificial lighting, around-the-clock availability of food or shiftwork. Such contradictory zeitgeber input promotes chronodisruption, *i.e.*, the perturbation of internal circadian rhythms, resulting in adverse health outcomes. While this phenomenon is well described, it is still poorly understood at which level of organization perturbed rhythms impact on health and wellbeing. In this review, we discuss different levels of chronodisruption and what is known about their health effects. We summarize the results of disrupted phase coherence between external and internal time vs. misalignment of tissue clocks amongst each other, i.e., internal desynchrony. Last, phase incoherence can also occur at the tissue level itself. Here, alterations in phase coordination can emerge between cellular clocks of the same tissue or between different clock genes within the single cell. A better understanding of the mechanisms of circadian misalignment and its effects on physiology will help to find effective tools to prevent or treat disorders arising from modern-day chronodisruptive environments.

## Introduction

**Figure 1.**
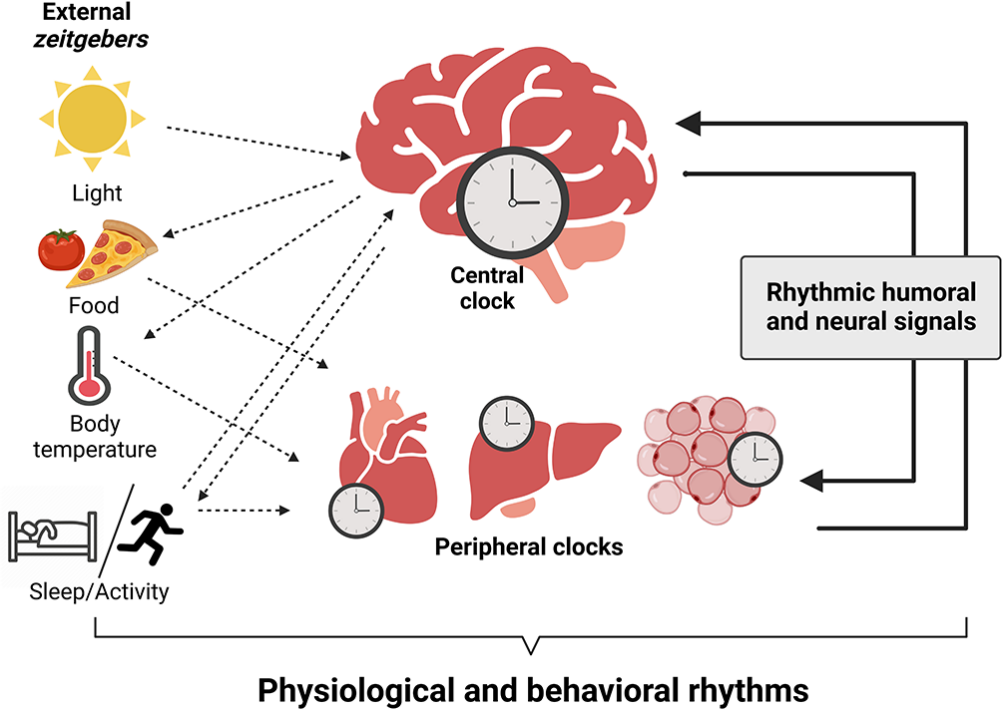
Organization of the circadian system in mammals. The primary *zeitgeber*, light, entrains the master pacemaker, the suprachiasmatic nucleus (SCN), with geophysical time. The SCN synchronizes subordinated central nervous system (CNS) and peripheral tissue clocks by humoral and neural signals and the temporal coordination of food intake, body temperature, and rest/activity cycles. Outputs of peripheral tissues feedback to clocks in the brain and stabilize circadian synchrony. Integration of external signals, collective output of tissue clocks, and rhythmic humoral and neural signals generates physiological and behavioral circadian rhythms. Figure created with BioRender.com.

Mammals possess a ubiquitously expressed circadian clock system with a master pacemaker located in suprachiasmatic nucleus (SCN), in the hypothalamus, driving physiological and behavioral rhythms. Such rhythms can be observed in, e.g., hormonal release, eating patterns, sleep behavior and body temperature ( Moore and Eichler, 1972; Ralph *et al.*, 1990; Sawaki *et al.*, 1984; Stephan and Zucker, 1972). Functional clocks have been found in the SCN but also numerous other tissues including liver, kidney, and adipose tissues ( Aschoff, 1965; Aschoff *et al.*, 1967; Balsalobre *et al.*, 1998; Lamia *et al.*, 2008; Yamazaki *et al.*, 2000; Yoo *et al.*, 2004). All circadian clocks share three common properties. First, circadian oscillators are self-sustained, i.e., they are capable of driving ~24-hour circadian rhythms in transcription and translation. Second, they preserve the same kinetics over a broad range of temperatures ( Barrett and Takahashi, 1995; Menaker and Wisner, 1983; Pittendrigh, 1954; Reyes *et al.*, 2008). Third, circadian oscillators can be synchronized (or *entrained*) by environmental cues, so called *zeitgeber*s (German for ‘time giver’) such as light and food ( Balsalobre *et al.*, 1998; Yoo *et al.*, 2004). Photic zeitgeber input synchronizes the SCN that in turn aligns the central nervous system (CNS) and peripheral-tissue clocks with each other and with external time *via* humoral and neural signals. Together, these give rise to rhythmic circadian output ( Figure 1). Single circadian oscillators have distinct period lengths, and it is thought that they must be synchronized to the 24-hour light/dark (LD) cycle to provide coherent rhythmic control over physiological processes such as maintaining temporal separation of chemically incompatible processes ( Honma *et al.*, 1998; Liu *et al.*, 2007; Nagoshi *et al.*, 2004; Welsh *et al.*, 1995). Modern lifestyles are often characterized by deregulated food intake rhythms, lack of exercise, disrupted sleep/wake patterns, and nocturnal light exposure. Such mismatch of zeitgeber signals is believed to lead to disruptions in the phase coherence between internal and external time and between different tissue clocks in a state termed *chronodisruption* ( Erren and Reiter, 2009, 2013; Kiehn *et al.*, 2017).

The circadian clock system regulates energy homeostasis, and dysregulation of circadian clock-metabolism crosstalk can seriously impact overall metabolic health ( Reinke and Asher, 2019). For example, eating at a biologically inappropriate phase results in impaired glucose tolerance and transient insulin resistance in a laboratory study ( Scheer *et al.*, 2009). Nighttime eaters gain more weight compared to non-nighttime eaters ( Gluck *et al.*, 2008). Increased light at night (LAN) exposure is associated with a higher incidence of obesity and metabolic syndrome ( McFadden *et al.*, 2014). Possibly, this may be provoked by light-mediated resetting of the SCN circadian clock and inhibition of melatonin release ( Boivin *et al.*, 1996; Zeitzer *et al.*, 2000). Additionally, in modern societies roughly 21% of employees work outside the regular working hours or in shifts, which has been associated with various adverse health outcomes ( Karatsoreos *et al.*, 2011; Parent-Thirion *et al.*, 2016; Streng *et al.*, 2022). Shift work is believed to affect the alignment of an individual’s behavioral cycle with both external and endogenous rhythms resulting in a loss of phase coherence between two or more circadian rhythms ( Boudreau *et al.*, 2013; Skene *et al.*, 2018). Numerous epidemiological studies have demonstrated a higher prevalence of various disorders in shift workers – from impaired mental health to metabolic syndrome, obesity, cardiovascular disease, autoimmune disorders, and cancer ( Chellappa *et al.*, 2020; Davis *et al.*, 2001; de Bacquer *et al.*, 2009; di Lorenzo *et al.*, 2003; Ellingsen *et al.*, 2007; Karlsson *et al.*, 2001; Kawachi *et al.*, 1995; Li *et al.*, 2022; Magrini *et al.*, 2006; Schernhammer *et al.*, 2003). Circadian misalignment can also be caused by travelling across several time zones leaving us with sleep problems, fatigue, cognitive impairments, and gastrointestinal issues summarized as *jetlag disorder.* During jetlag, the body’s internal time is misaligned to local geophysical time, and the circadian clock network needs some time to entrain to the new time zone ( Diekman and Bose, 2018; Kiessling *et al.*, 2010; McGuckin *et al.*, 2014; Roach and Sargent, 2019; Wright *et al.*, 1983).

## Levels of organization of the mammalian circadian clock system

The SCN constitutes a network of interconnected neural clocks with distinct periods *in vitro* ( Honma *et al.*, 2004; Welsh *et al.*, 1995). However, a robust circadian output is observed probably arising from strong inter-cellular coupling within the SCN. Non-SCN tissue clocks show fast dampening of circadian oscillations *in vitro* likely due to weak inter-cellular coupling and the high dependence on systemic signals to keep synchrony ( Yoo *et al.*, 2004). Coupling can be observed on different levels such as systemic (inter-tissue) and intra-tissue which can be further subdivided into inter-cellular and molecular. All contribute to the generation or maintenance of coherent rhythms in behavior and physiology.

The SCN synchronizes the clock network via numerous signals. Vice versa, rhythmic signals from the periphery feedback to the SCN to stabilize and fine-tune the clock system. This type of crosstalk occurring between different tissue clocks is known as *systemic coupling* ( Pilorz *et al.*, 2020). The autonomic nervous system and the endocrine system are important routes utilized by SCN to deliver humoral and neural signals to peripheral tissues. Thereby, the SCN regulates changes in the blood supply, kidney filtration rates, hormone secretion, sensitivity for hormones, and insulin sensitivity in a time-dependent manner ( Buijs *et al.*, 1999, 1993; Cailotto *et al.*, 2005; Cui *et al.*, 2001; Dai *et al.*, 1997; Deering and Coote, 2000; Kalsbeek *et al.*, 1993; Kalsbeek and Strubbe, 1998; Vujovic *et al.*, 2015). The best studied circadian hormones are glucocorticoids and melatonin, and both act as systemic synchronizing signals ( Cheifetz, 1971; de Kloet and Sarabdjitsingh, 2008; Klemcke *et al.*, 1989; Lincoln *et al.*, 1982; Oster *et al.*, 2006; Perlow *et al.*, 1981; Redman *et al.*, 1983). On the other hand, systemic signals derived from peripheral tissues reach the SCN for a proper synchronization of physiological rhythms ( Balsalobre *et al.*, 2000; Buijs *et al.*, 1999; Gerber *et al.*, 2013; Guo *et al.*, 2005; Oster *et al.*, 2006; Redman *et al.*, 1983). For this reason, various hormone receptors are present in the SCN modulating and fine-tuning internal circadian timing. Intriguingly, the hepatokine fibroblast growth factor 21 (FGF21), a starvation signal secreted from the liver into the blood stream, can affect the SCN, thereby modulating several physiological functions ( Bookout *et al.*, 2013). Another example, leptin, an adipocyte derived peptide, conveys the metabolic state of peripheral tissues to the SCN. Interestingly, leptin receptor deficient rats show disrupted circadian rhythms of food intake ( Li *et al*., 2012). The stomach-derived peptide hormone ghrelin can modulate food anticipatory activity through its action on the mediobasal hypothalamus (MBH) ( Merkestein *et al.*, 2014; Wang *et al.*, 2018). Thus, systemic coupling mechanisms are crucial to efficiently synchronize circadian rhythms across the entire body.

On tissue level, one can discriminate between inter-cellular and molecular coupling. For inter-cellular coupling, single cells within a tissue communicate with each other using various signaling mechanisms, thereby, creating internal synchrony across the organ. Coupling between different cells of the same tissue is best described for the SCN. Here, gap junctions, paracrine signals, chemical synapses, and electrical signaling are used to keep SCN cell rhythms synchronized and aligned with each other ( Maywood *et al.*, 2011; Rash *et al.*, 2007; Yamaguchi *et al.*, 2003). Tetrodotoxin-mediated inhibition of neurotransmission results in reduced amplitudes of circadian clock gene expression ( Yamaguchi *et al.*, 2003). Strong inter-cellular coupling in the SCN results in robust rhythms which can also compensate genetic clock disruption for which peripheral tissues are much more sensitive ( Liu *et al.*, 2007). In line with this, SCN explants show circadian oscillations for weeks whereas circadian oscillations diminish in peripheral tissue explants after some days suggesting much weaker inter-cellular coupling ( Yoo *et al.*, 2004). However, hepatocyte clocks in SCN ablated mice show fast and stable entrainment to daytime feeding indicating that peripheral tissue clocks have to be coupled to a certain extent ( Saini *et al.*, 2013). It remains elusive whether the synchronized circadian oscillations in liver arise through the integration of systemic signals or by coupling on the tissue level. Hepatocyte cell culture experiments suggest that there is weak coupling between the cells since desynchronization over time is slower than in a simulation of a model where no coupling is assumed. Coupling in the periphery is weak and locally more restricted compared to what is shown for the SCN ( Guenthner *et al.*, 2014), and the underlying mechanisms are largely unknown.

Molecular coupling within a rhythmic cell is established through interlocked transcriptional- translational feedback loops (TTFLs) regulating rhythmic transcription and post-translational modifications controlling the stability of clock proteins ( Buhr and Takahashi, 2013). Within each rhythmic cell, the molecular clock has a normal phase distribution. The new transcriptional cycle of the molecular clock starts once brain and muscle ARNT like protein 1 (BMAL1): circadian locomotor output cycles kaput (CLOCK) heterodimers bind to *E-box* promotor elements in *period1–3* ( *Per1–3*) and *cryptochrome1/2* ( *Cry1/2)* genes inducing their transcription in the morning ( Kume *et al.*, 1999; Shearman *et al.*, 1997). Later, towards the night, PER and CRY heterodimers are translocated to the nucleus where they repress their own transcription by inhibiting binding of BMAL1:CLOCK to *E-boxes* ( King *et al.*, 1997; Konopka and Benzer, 1971; Reddy *et al.*, 1984; van der Horst *et al.*, 1999; Zheng *et al.*, 1999). Beside the *E-box* elements, other response elements such as *D-boxes* and retinoic acid receptor-related orphan receptors elements ( *ROREs*) are modulating and stabilizing circadian oscillations ( Akashi and Takumi, 2005; Mitsui *et al.*, 2001; Nakajima *et al.*, 2004; Ohno *et al.*, 2007; Preitner *et al.*, 2002; Sato *et al.*, 2004; Ueda *et al.*, 2002; Yamaguchi *et al.*, 2000). *D-boxes* are bound among others by the transcriptional repressor nuclear factor interleukin-3-regulated protein (NFIL3, also known as E4BP4) and the transcriptional activator *D-box* binding protein (DBP) modulating the expression of, e.g., *Per1–3*, repressive reverse-erythroblastosis virus α/β ( *Rev-Erbα/β*), and activating retinoic acid receptor-related orphan receptors ( *Rorα/β/γ*) ( Mitsui *et al.*, 2001; Ohno *et al.*, 2007; Ripperger and Schibler, 2006; Yamaguchi *et al.*, 2000). Repressing REV-ERB α/β and activating RORα/β/γ proteins compete for binding to ROREs modulating the expression of *Bmal1*, *Clock* and *Cry1* ( Guillaumond *et al.*, 2005; Nakajima *et al.*, 2004; Preitner *et al.*, 2002; Sato *et al.*, 2004; Ueda *et al.*, 2002). The interlocked feedback-loops result in ~24- hour oscillation of gene expression and repression. Importantly, such oscillations are further stabilized and fine-tuned by chromatin remodeling, post-transcriptional, and post-translational modifications impacting the stability of distinct clock mRNAs/proteins ( Doi *et al.*, 2006; Duong and Weitz, 2014; Gallego and Virshup, 2007; Grimaldi *et al.*, 2007; Nakahata *et al.*, 2008; Grimaldi *et al.*, 2009; Kojima *et al.*, 2011; Preußner *et al.*, 2014). Together, these lead to a stable phase relationship/coherence between the different genes. In general, expression of *Bmal1* is almost anti-phasic to the expression of *Per2, Nr1d2* and *Cry2* (10–12 h), whereas expression of *Nr1d1* is only phase-delayed by 6–10 h. Interestingly, the peak expression of *Bmal1* and *Cry1* differs only between 2–4 h ( Mure *et al.*, 2018). Of note, the phase relationship of the clock genes to each other is tissue- and species-specific ( Harbour *et al.*, 2014; Korenčič *et al.*, 2014; Mure *et al.*, 2018; Pett *et al.*, 2018; Yeung *et al.*, 2018). Moreover, there is a stable phase relationship of single-tissue clocks to each other. In general, non-SCN tissues are phase-delayed compared to the SCN. Adrenal gland clocks show a small phase delay compared to those in the liver, whereas liver, adipose tissue, and muscle are similarly phased ( Korenčič *et al.*, 2014; Mure *et al.*, 2018; Yang *et al.*, 2006). Since all tissue clocks contribute to generate coherent circadian rhythms, the phase coherence within the cell, between the cells and among the tissues is potentially important to maintain homeostasis.

Genetic mutations or knockouts (KO) of circadian clock genes lead to the development of various diseases ranging from sleep disorders to cardiovascular, mental, and metabolic deteriorations ( Dibner and Schibler, 2015; Kiehn *et al.*, 2017; Valenzuela *et al.*, 2016). In recent years, evidence has been accumulated on the physiological relevance of distinct tissue clocks. Liver-specific *Bmal1* KO results in abolished rhythms in glucose regulatory genes creating problems in the maintenance of blood glucose levels especially during fasting periods ( Lamia *et al.*, 2008). Elevated levels of blood lipids were observed in mice with hepatocyte-specific ablation of *REV-ERBα/β* ( Bugge *et al.*, 2012; Cho *et al.*, 2012) *.* Skeletal muscle clock regulates glucose metabolism. Mice lacking a functional muscle clock show defective insulin-stimulated glucose uptake attributed to impaired glucose transporter 4 (GLUT4) translocation to plasma membrane ( Dyar *et al.*, 2014; Harfmann *et al.*, 2016). In adipose tissue, circadian clocks regulate several metabolic processes in a time-of-day dependent manner. Adipocyte-specific *Bmal1* KO results in obesity and increased rest-phase food intake probably related to altered fatty acid signaling to the hypothalamus ( Paschos *et al.*, 2012). In heart, genetic ablation of *Bmal1* results in perturbed systolic function and abnormal glucose utilization ( Young *et al.*, 2014). Lastly, adrenal cortex-specific *Bmal1* KO mice display dampened glucocorticoid and locomotor activity rhythms ( Dumbell *et al.*, 2016; Son *et al.*, 2008). Of note, local tissue clocks are not sufficient to drive the local transcriptome independently ( Koronowski *et al.*, 2019). Altogether, these studies emphasize the importance of specific tissue clocks, but they also show that coherence among different tissue rhythms is important to maintain whole body homeostasis.

## From a circadian-resonant to a chronodisrupted health state

**Figure 2.**
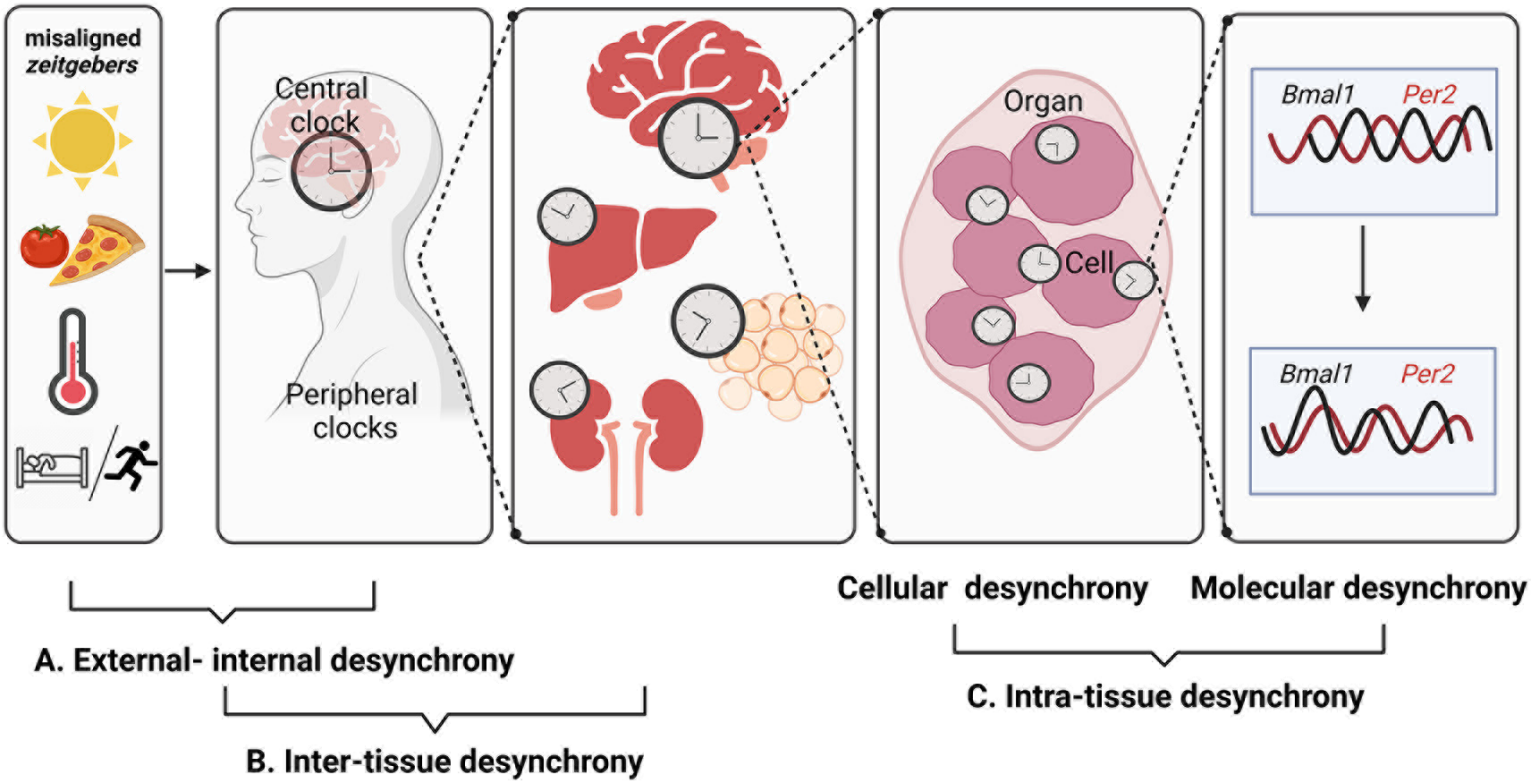
Different levels of circadian desynchrony. Misaligned *zeitgebers* like artificial lighting conditions, mistimed food intake, or activity in the rest phase create dissonance between the internal circadian timing system and geophysical time – a state somewhat vaguely termed as *chronodisruption.* Desynchrony of circadian rhythms can occur at various levels – from misalignment of internal and external time (as during jetlag) to alterations in the coordination of different clock gene expression rhythms at the cellular level. It is still poorly understood how the different levels of circadian desynchrony contribute to the adverse health effects of chronodisruption. Figure created with BioRender.com.

Circadian clocks throughout our body integrate external and internal rhythmic signals to drive coordinated rhythmic physiological and behavioral outputs. When these inputs are appropriately aligned, the body is in a resonant or synchronous state which enhances resilience to pathogenic insults and thus, stabilize health and wellbeing. However, when zeitgeber input is perturbed, e.g., the occurrence of temporal stimuli is temporally shifted or the duration is altered, chronodisruption may emerge. With regard to different zeitgebers, the LD cycle is considered as the most potent zeitgeber for entraining circadian and seasonal rhythms in most species. In real life, a misalignment of geophysical time and circadian rhythms is observed after rapidly crossing different time zones. This provokes jetlag disorder with disrupted sleep/wake cycles, fatigue and cognitive impairments until the internal clock system is entrained to the new geophysical time. While for the general population jetlag occurs only once in a while, which has little persisting health effects, long-distance flight personnel experiences repeated jetlag which may cause long-term impairments. Most people in modern societies, on the other hand, are exposed to artificial light during natural dark periods. Increasingly more people are experiencing LAN during work in shifts (reviewed in detailed by Touitou *et al.*, 2017). Shift workers commonly have inefficient and poor quality sleep causing fatigue ( Paech *et al.*, 2010). Moreover, forced activity and sleep deprivation in the normal rest phase strongly impacts the immune system ( Irwin *et al.*, 2006), and shift workers are more likely to develop chronic medical conditions like cardiovascular diseases ( Ha and Park, 2005), metabolic syndrome ( Cheng *et al.*, 2021), psychological disorders (reviewed by P. Cheng & Drake, 2018) and others. Simulations of shift work and abnormal LD cycles in laboratory settings are used to investigate the mechanisms of chronodisruption and will be described in detail in the next sections. Usually feeding and social behavior are also almost exclusively scheduled during active phases of the circadian cycle. They serve as potent entraining stimuli to some CNS and peripheral tissue clocks. Availability of food round-the clock and energy requirement during shiftwork leads to mistimed food intake in humans that manifests into various chronic metabolic diseases including obesity and type-2 diabetes. Tissues clocks that are strongly influenced by feeding signals, e.g. liver or kidney, can be uncoupled from the SCN inducing inter-tissue desynchrony ( Damiola *et al.*, 2000; Hara *et al.*, 2001; Stokkan *et al.*, 2001). All of the above described real-life situations result in chronodisruption which promotes metabolic impairments but also increases the prevalence for major depression, cardiovascular disease, autoimmune disorders, and certain cancers ( Davis *et al.*, 2001; de Bacquer *et al.*, 2009; di Lorenzo *et al.*, 2003; Ellingsen *et al.*, 2007; Erren and Reiter, 2009, 2013; Karlsson *et al.*, 2001; Kawachi *et al.*, 1995; Li *et al.*, 2022; Magrini *et al.*, 2006; Schernhammer *et al.*, 2003). Although the concept of chronodisruption is established, its pathogenic mechanism remains largely elusive. Chronodisruption can occur at different levels, i.e., (1) a mismatch of external time and internal circadian rhythms ( *external-internal desynchrony*), (2) phase incoherence between different tissue clocks ( *inter-tissue desynchrony*), (3) phase incoherence between cells of the same tissue ( *cellular desynchrony*) and, (4) phase incoherence at the molecular level ( *molecular desynchrony*) ( Figure 2). In the following sections we will describe these different levels of desynchrony and outline what is known about their physiological consequences.

## Desynchrony between external and internal rhythms

Misalignment between zeitgebers and internal circadian rhythms evokes chronodisruption ( Figure 2). This state is often caused or enhanced by perturbed zeitgebers such as phase-shifted LD cycles, activity in the inactive phase (shift work), or mistimed food intake, all of which have been suggested to promote metabolic and psychological ailments.

Mismatch between geophysical time and internal biological timing, as observed in jetlag conditions, causes physiological behaviors like sleep, hunger and defecation to occur according to internal timing at non-regular times of the day. However, people in aviation industries who are exposed to repeated jetlag report chronic sleep-inefficiency associated physiological (reviewed in Arendt and Marks, 1982) and cognitive health problems ( Cho *et al.*, 2000). Simulated chronic jetlag conditions induce obesity in mice ( Oike *et al.*, 2015). However, body weight gain is observed only in advanced but not in delayed LD cycles ( Casiraghi *et al.*, 2016). Chronic jetlag retains oscillations, but induces phase shifts in clock gene expression rhythms in SCN and liver of mice ( Iwamoto *et al.*, 2014). In laboratory settings, rodents are exposed to non-24-hour LD cycles (T-cycles) to investigate the mechanisms of entrainment. Chronic exposure to short T-cycles (4 h light: 4 h darkness (4:4 LD)) — but not long T-cycles (18:18 LD) — decreases life span. Interestingly, clock deficient mice show no differences in mortality rates under long and short T-cycles. The authors suggest that the combination of specific LD cycles and perturbations of the circadian clock network results in impaired homeostatic regulation impacting longevity ( Park *et al.*, 2012). In mice, T-cycles, both 11.25:11.25 LD and 13.5:13.5 LD, reduce energy efficiency evidenced by higher food intake despite no body weight gain. Animals increase their food consumption during the light phase. Thereby, mice predominantly utilize carbohydrates to fuel the body which hints to an impaired metabolic homeostasis ( West *et al.*, 2017). Under 11:11 LD cycles, rats show dyslipidemia and lowered expression levels of proteins critical for insulin signaling as well as increased levels of enzymes involved in gluconeogenesis in liver indicating metabolic disruptions induced by forced desynchrony ( de Oliveira *et al.*, 2019). Non-24-hour LD cycles result in abolished rhythmicity in heart rate and blood pressure ( Molcan and Zeman, 2017; West *et al.*, 2017). In humans, circadian misalignment evokes an overall increase in blood pressure ( Morris *et al.*, 2016; Scheer *et al.*, 2009). Of note, the loss of rhythmic LD cycles as experienced during LAN or constant light (LL) conditions also impacts circadian outputs such as food intake patterns, energy expenditure and corticosterone rhythms promoting elevated body weight, metabolic impairments and depressive-like behavior ( Coomans *et al.*, 2013; Fonken *et al.*, 2012, 2010; Tapia-Osorio *et al.*, 2013).

Shift workers are forced to be active during their normal rest phase. Such work regimes are often accompanied by food consumption at inappropriate times. Thus, shift workers are simultaneously exposed to various chronodisrupters such as LAN, mistimed food intake and mistimed behavioral rhythms. Such employees show dampened cortisol and testosterone rhythms ( Touitou *et al.*, 1990). Furthermore, shift workers exhibit higher triglyceride and lower HDL (high density lipoprotein) cholesterol levels and higher abdominal obesity than regular day-time workers ( Karlsson *et al.*, 2003). Night shift work can be simulated in rodents by forced activity in the normal resting phase. This shifts activity and food intake into the resting phase. Consequently, daily blood glucose rhythms are abolished, triglyceride rhythms are reversed and corticosterone levels are transiently increased ( Salgado-Delgado *et al.*, 2008). In laboratory studies, shift work can be simulated by imposed 28-hour days in humans. In such conditions, sleep and food intake patterns are 12 h out of phase from the habitual times within a few days. On these (misaligned) days, participants exhibit completely reversed cortisol rhythms and increased arterial pressure. In addition, they show impaired metabolic homeostasis with increased blood glucose despite increased insulin levels compared to the circadian aligned baseline day ( Scheer *et al.*, 2009). Interestingly, daytime-restricted food consumption prevents the dissociation of body temperature rhythms, a measure of the central clock, and peripheral glucose rhythms. In turn, this prevents detrimental outcomes in glucose tolerance especially in the biological morning in the 28-hour shift work protocol ( Chellappa *et al.*, 2021). The studies show that already short-term chronodisruption generates adverse metabolic and cardiovascular effects. In addition to metabolic and psychological repercussions, far more detrimental effects of chronodisruption induced by shiftwork are carcinogenesis and malignancy. Numerous studies have shown cancer as a consequence of such a work regime ( Davis *et al.*, 2001; Mazzoccoli *et al.*, 2011; Schernhammer *et al*., 2003; Viswanathan *et al.*, 2007). However, a recent meta-analysis shows overall no significant association between the two ( Dun *et al.*, 2020). The meta-analysis considers factors like geophysical region, time and gender differences to compare data between different cohorts; however, factors like type of shift work, food intake habits and general health condition of the individual may also play a role in the cancerogenic effects of shift work. Not only shift workers, but even larger parts of the working force face discrepancy in their sleep/wake cycles between workdays and free days imposing a state termed *social jetlag.* This regular shift in sleep/wake phasing likely represents a condition of chronic circadian misalignment. People with strong social jetlag show higher cortisol levels and increased resting heart rates ( Rutters *et al.*, 2014). Social jetlag is also associated with increased body mass index and metabolic syndrome ( Roenneberg *et al.*, 2012; Parsons *et al.*, 2015). Unhealthy obese subjects show elevated levels of the inflammatory marker C-reactive protein and the obesity-related biomarker glycated hemoglobin with increased social jetlag ( Parsons *et al.*, 2015). These data show that chronodisruption impairs metabolic homeostasis and contributes to aggravation of the medical conditions.

Food intake, being an important zeitgeber especially for peripheral tissue clocks, disturbs internal resonance of rhythms when occurring at the wrong time-of-day. On the long-term, this may result in obesity and other metabolic disorders. Daytime restricted feeding in nocturnal mice uncouples the liver clock from the SCN ( Damiola *et al.*, 2000; Hara *et al.*, 2001; Stokkan *et al.*, 2001). Food intake only during the light phase also phase-shifts the acrophase of genes involved in lipid homeostasis and bile acid metabolism which may cause adverse metabolic outcomes ( Cui *et al.*, 2022). High-caloric food is shown to disrupt daily food intake patterns and rhythms in clock as well as metabolism related genes. Such disruption can be prevented when the high-caloric diet is only available during the night in nocturnal mice. This intervention also reduces metabolic diseases like hepatic steatosis and hypercholesterolemia observed in *ad libitum* fed mice ( Chaix *et al.*, 2014).

In modern societies, several zeitgebers are misaligned to each other and to the endogenous circadian clock. When mice are subjected to a combined 14:14 LD with a 12:12 fasting/feeding (FF) protocol they become dynamically exposed to aligned and misaligned zeitgeber conditions. Surprisingly, mice transiently show weight gain and impaired glucose tolerance on the day of aligned zeitgeber input (i.e., when feeding coincides with the dark phase) compared to the day of misaligned zeitgeber input (feeding during the light phase). Such zeitgeber misalignment further evokes a change in the phasing of different tissue clocks ( Heyde and Oster, 2019). In conclusion, misalignment between internal and external rhythms is associated with alterations in metabolic and psychological states and may promote obesity, diabetes, cardiovascular diseases, depression, anxiety, and cancer.

## Inter-tissue desynchrony

Desynchrony caused due to external-internal misalignment can impinge further than just the systemic level by inducing phase incoherence between different tissue clocks and circadian tissue outputs. Tissues differ in their susceptibility to external and hormonal signals from each other. Thus, they adapt to changed input rhythms at a different pace inducing misalignment between tissue clocks and rhythms – a state termed as inter-tissue desynchrony.

Circadian homeostasis is maintained by communication and synchronization between and among central and peripheral clocks. Ablation of the SCN clock eliminates diurnal variation in clock gene expression in peripheral tissues including liver, kidney, heart, skeletal muscle, and spleen. Parabiosis of SCN-lesioned and intact animals re-induces day-night variation in SCN-lesioned mice in liver and kidney but not in heart, skeletal muscle and spleen. This emphasizes the differential dependence on blood-borne signals of distinct tissue clocks ( Guo *et al.*, 2005). However, recent studies observed that in LD the SCN clock is dispensable for clock network synchronization but becomes necessary under constant darkness (DD) conditions. Animals with an ablated SCN clock show internal desynchrony, i.e., phase incoherence between different tissue clocks, within a few days under DD conditions ( Husse *et al.*, 2011; Izumo *et al.*, 2014; Kolbe *et al.*, 2019; van der Vinne *et al.*, 2018). However, effects of internal desynchrony on body weight seem to be dependent on the Cre driver line used ( Kolbe *et al.*, 2019; van der Vinne *et al.*, 2018). Of note, internal desynchrony and weight gain are prevented by maintaining the animals on time-restricted feeding schedules under DD conditions highlighting the importance of food intake timing for peripheral clock synchronization ( Kolbe *et al.*, 2019).

Many studies use constant light (LL) or repeated jetlag conditions to investigate the effects of abnormal light cycles on different tissue clocks. LL conditions not only weaken the central clock but also disrupt phase alignment between different tissues. Mice subjected to LL show immediate dampening in the amplitude of SCN rhythms ( Coomans *et al.*, 2013). LL housing maintains rhythmic PER2 oscillations in liver and kidney but distinctly reduces amplitude and broadly distributes phases of its rhythm. In submandibular gland, this rhythm is lost in a significantly higher number of animals ( Hamaguchi *et al.*, 2015). On transcriptional level, the liver and colon display abolished diurnal expression of clock genes ( Polidarová *et al.*, 2011). In addition, to clock outputs, LL eliminates rhythmicity of numerous genes involved in lipid metabolism in liver and white adipose tissue (WAT). Decreases in amplitude and average levels of gene expression are more pronounced in the liver compared to WAT, indicating higher sensitivity of metabolic and clock gene transcription in the liver to constant light conditions ( Yamamuro *et al.*, 2020). Upon jetlag, distinct tissue clock genes display differences in entrainment speed to the new LD schedule. Here, the misalignment caused is temporary and is resolved when tissue clocks re-align with the new LD cycle. SCN and adrenal rapidly adapt to the changed lighting schedule whereas liver and kidney re-entrain at a slower pace, with the pancreas clock being the slowest ( Kiessling *et al.*, 2010). PER1 oscillations in the arcuate nucleus quickly entrain upon a phase-delaying jetlag whereas the paraventricular nucleus (PVN) and pineal gland are faster to re-entrain upon a phase-advancing jetlag in in vitro studies ( Abe *et al.*, 2002). These studies indicate that it is likely that jetlag induces internal desynchronization not only between central and peripheral clocks but also between clocks within the central nervous systems. The sleep/wake cycle is one of the behavioral outputs driven by the SCN. Two weeks of timed-sleep restriction, mimicking human night shiftwork, have only moderate effects on the levels of clock gene expression in the SCN but markedly change expression levels and phasing in the liver. Moreover, circadian liver transcriptome data show pronounced changes especially in glucose metabolism related genes which results in impaired performance in gluconeogenesis and increased glycogen storage in the beginning of the dark phase. Time-restricted feeding to the normal active phase is able to prevent the effects of time-restricted sleeping on hepatic clock gene expression and metabolic outcomes ( Barclay *et al.*, 2012).

Peripheral clocks show tissue-specific pace in feeding-induced phase resetting. Upon daytime restricted feeding, phases of the clocks in liver, kidney, heart, lungs and pancreas in mice are transiently misaligned to each other. However, all these tissues entrain to the new feeding schedule within a week ( Damiola *et al.*, 2000; Hara *et al.*, 2001; Opperhuizen *et al.*, 2016; Stokkan *et al.*, 2001). The dorsomedial hypothalamus also entrains to daytime restricted feeding ( Verwey and Amir, 2011). The SCN clock, however, stays aligned to the LD cycle ( Damiola *et al.*, 2000; Hara *et al.*, 2001; Opperhuizen *et al.*, 2016; Stokkan *et al.*, 2001). In contrast, clock gene rhythms are abolished in lateral hypothalamus, skeletal muscle and arcuate nucleus in rats fed during the light period ( Opperhuizen *et al.*, 2016; Wang *et al.*, 2017). The studies show that feeding time has differential effects on SCN and non-SCN CNS as well as peripheral clocks, thus, weakening inter-tissue phase coherence — which likely interferes with the maintenance of metabolic homeostasis.

In addition to the mistiming of food intake, changes in diet composition can induce internal desynchrony. High-fat diet (HFD) feeding is known to disrupt behavioral and physiological rhythms, but the mechanisms are still not fully understood. It is likely that internal desynchronization of circadian rhythms plays an important role. Long-term HFD feeding alters clock gene expression rhythms in liver and adipose tissue ( Kohsaka *et al.*, 2007; Yamamuro *et al.*, 2020). Minimal effects on clock gene rhythms were observed in MBH, a center which controls hunger and satiety, and the medial prefrontal cortex, a region involved in cognitive functions ( Kohsaka *et al.*, 2007; Tognini *et al.*, 2020). Of note, HFD reduces the total number of oscillating genes, significantly advances the acrophase and dampens the amplitude of rhythmic genes, but not clock genes, in the SCN demonstrating that the SCN is susceptible to food signals ( Tognini *et al.*, 2020). The liver clock is not only susceptible to feeding time but also to food composition. *In vitro* studies confirm a high susceptibility of the liver clock to food-related signals whereas other tissue clocks such as lung, pituitary and arcuate complex are less receptive ( Pendergast *et al.*, 2013). Food related signals which modulate clock resetting include insulin ( Chaves *et al.*, 2014; Crosby *et al.*, 2019; Sato *et al.*, 2014; Sun *et al.*, 2015; Tahara *et al.*, 2011), oxyntomodulin ( Jorgensen *et al.*, 2007; Landgraf *et al.*, 2015), and many others (reviewed in Reinke and Asher, 2019). Additionally, glucose, amino acids and other metabolites can also phase-shift or entrain circadian clocks (reviewed in Froy, 2007). As such, it can be speculated that re-entrainment of a distinct tissue clock is dependent on its susceptibility to clock modulators. Altogether, the timing of food intake and food composition can rapidly lead to inter-tissue phase incoherences which might be transient or permanent.

Under diverging zeitgeber input (14:14 LD cycle combined with a 12:12 FF cycle), liver and epididymal WAT clocks align with the FF schedule whereas adrenal and SCN tissue clocks are highly impacted by the extended LD cycle. Thus, animals suffer from internal desynchrony between distinct tissue clocks and circadian output. Interestingly, mice do not gain weight but show circulating body weight changes dependent on the transient state of zeitgeber (mis)alignment ( Heyde and Oster, 2019). Such internal desynchrony is already observed after four days of 14:14 LD/12:12 FF conditions and is less pronounced when feeding is restricted to the dark phase under 14:14 LD conditions ( Heyde and Oster, 2022).

Taken together, changes in the external environment or (forced) changes in the behavioral cycle result in phase incoherences between different oscillators which may cause disruption of rhythmic output eventually resulting in metabolic impairments. However, more studies investigating the effect of phase incoherences between certain tissues are needed to fully elucidate the physiological consequences.

## Intra-tissue desynchrony

Tissues contain numerous cell types with each cell containing an autonomous molecular clock. Synchronized cells give rise to overt circadian rhythms on the tissue level. Cells integrate several signals to produce coherent rhythms. Phase incoherences of single cellular clocks within a tissue cause intra-tissue desynchrony. Moreover, at the single-cell level the phasing of distinct clock gene rhythms to each other can be disrupted giving rise to molecular desynchrony ( Figure 2). Current standard techniques face limitations in distinguishing between cellular and molecular levels of chronodisruption. Thus, in this section we describe both levels together as intra-tissue desynchrony.

With regards to desynchrony caused between different cells the best-studied tissue is the SCN. The SCN consists of two major regions with light responsive neurons in the ventrolateral and light unresponsive ones in the dorsomedial part ( Van den Pol, 1980). Abrupt delaying or advancing of LD rhythms rapidly causes incoherence of synchronous rhythms of clock genes between the two SCN regions. Clock genes in the ventrolateral SCN are quick to show large shifts compared to the dorsomedial region which shifts slower and takes a longer time to resynchronize to the new lighting schedule ( Nagano *et al.*, 2003). Shifts in lighting conditions also disrupt phase relation, i.e., peak phase differences between dorsal and ventral parts of the SCN. After a phase-delaying shift in the LD cycle, peak phase differences between the regions increase but return to pre-phase shift conditions within three days. In contrast, a phase-advancing shift in the LD cycle leads to reversed phase relations between the regions and the pre-shift state is reached only after six days ( Nakamura *et al.*, 2005). A recent study with imposed delays in the LD cycle investigated *Per2* expression at a single-cell level throughout the SCN. The ventral part contained about 40% fast phase-shifting and 60% slow phase-shifting neurons while the dorsal SCN almost exclusively comprised slow-shifting neurons ( van Beurden *et al.*, 2022).

As mentioned above, phase incoherence can also occur at the level of the molecular clock work itself. In mice, in the SCN *Per1* and *Per2* rhythms more rapidly react to LD shift advances compared to *Cry1.* Whereas all three genes react rapidly to LD delays ( Reddy *et al.*, 2002). Similar transient dissociation is observed between *Per1* and *Bmal1* rhythms in mice exposed to a single light pulse during DD conditions. *Per1* is instantaneously phase-delayed together with activity onset while *Bmal1* shifts more gradually in parallel with activity offset ( Ono *et al.*, 2017).

Desynchrony within clock gene expression is also observed in peripheral tissue clocks. HFD attenuates amplitude of clock gene profiles in MBH, adipose and liver tissue. In the MBH, although *Per2* and *Bmal1* show robust rhythms, *Clock* RNA expression is completely abolished. In contrast, *Clock* is diurnally rhythmic in both fat and liver but with altered amplitude. Additionally, *Bmal1* is attenuated throughout the 24-hour day, but *Per2* shows decreased amplitudes only in the dark phase ( Kohsaka *et al.*, 2007). Notably, not only incoherence in phasing of clock genes but also differences in amplitude of their rhythm may contribute to desynchrony. However, it has to be investigated whether changes in amplitude result from impaired clock gene expression on a single-cell level or broader averaging over phase distributed cells. Differential pace of entrainment is observed for distinct clock genes upon jetlag conditions. In the SCN, somatosensory cortex and adrenals, *Per1* and *Per2* rapidly entrain to the new LD schedule whereas entrainment of *Dbp* and *Nr1d1* is slower. Interestingly, in the liver, *Per1* and *Dbp* re-entrainment is slower compared to *Per2* expression rhythms. *Bmal1* shows the slowest pace of entrainment in all tissues. Of note, in the pancreas, *Nr1d1* is quickly re-adjusted while *Per1* and *Per2* are slower ( Kiessling *et al.*, 2010) *.* Under zeitgeber intervention conditions, a higher degree of phase incoherence between clock gene expression peaks is observed under LD-28/FF-28 compared to LD-28/FF-24 conditions in liver, adrenal, and epididymal WAT ( Heyde and Oster, 2022) *.*

Exploring circadian characteristics on the single-cell level within a tissue is technically challenging and not thoroughly studied yet. In contrast, more evidence on phase incoherence between different clock genes within a tissue is arising. Both levels of intra-tissue desynchrony might impinge on the circadian output of the tissue affecting health, but more studies are needed to elucidate the specific impact.

## Therapeutic approaches and future directions

Circadian clock network coordination is disrupted by alterations in zeitgeber input rhythms. Numerous studies show that such perturbation may result in metabolic, mental, and cardiovascular impairments. It is important to consider that these ailments arise from circadian misalignment which occurs at different levels of organization. However, causality has to be proven. Disruption of circadian rhythms usually emerge due to misalignment between external time cues and internal time. Such misalignment promotes internal desynchrony between distinct tissue clocks. Although inter-tissue desynchrony is an established concept, experimental evidence is still sparse and, therefore, the physiological outcomes are not well understood. It can be speculated that desynchrony between cellular clocks of the same tissue contributes to dampening of circadian tissue output, which consequently impacts homeostasis. Again, so far this has not been proven directly. Finally, it remains elusive whether such perturbed circadian output is caused by impaired rhythmic transcription and translation at the cellular level or by phase incoherence between cells within the tissue.

It will be important to understand the mechanisms and effects of internal desynchrony at different levels to develop targeted therapeutic approaches that can prevent chronodisruption-associated diseases. Differentiating the physiological impact of desynchrony on each level may be technically challenging and good experimental paradigms dissecting the different forms of desynchrony are still sparse. As one example, the development of transgenic mice harboring luciferase reporters coupled to a clock gene promotor enabled researchers to investigate effects of zeitgeber intervention in real-time in freely moving animals ( Ono *et al.*, 2015; Saini *et al.*, 2013; Yamaguchi *et al.*, 2016). Recently, fluorescent clock-reporters were used to track circadian oscillation in SCN neuronal sub-populations *in vivo* ( Mei *et al.*, 2018). However, so far it is not possible to study changes on single-cell levels *in vivo.* *In vitro*, this is overcome by introducing two reporters. Here, one fluorescent clock-reporter gives information on the phase of the clock while the second fluorescent reporter is used to track single cells ( Manella *et al.*, 2021). Future developments in this direction might enable us to study the impact of zeitgeber input on single cells *in vivo* and may help in understanding the physiological effects.

To date, therapeutical approaches to increase internal synchrony are limited and not targeted but instead impact clocks throughout the body which may not in all cases be beneficial. Future studies should investigate whether modulation or weakening of distinct tissue clocks may be beneficial under chronodisruptive zeitgeber conditions. First findings suggest that weakening of the SCN clock or intra-tissue coupling might be beneficial for re-entrainment to shifted LD cycles; however, whole-body homeostasis was not studied ( An *et al.*, 2013; Yamaguchi *et al.*, 2013; Pilorz *et al.*, 2014). Bright-light therapy, affecting the SCN clock, reduces the misalignment of geophysical time and internal rhythms. It is shown to improve circadian rhythms and poor sleep quality in elderly, Alzheimer patients and people with major depression ( Yamadera *et al.*, 2000; Terman *et al.*, 2001; Neikrug *et al.*, 2012; Lam *et al.*, 2016; Rubiño *et al.*, 2020). Humoral signals impact on specific tissue clocks. However, we know too little about how the different tissue clocks are synchronized. Melatonin increases sleep quality and can entrain blind people to a 24-hour day ( Hack *et al.*, 2003; Redman, 1997; Sack *et al.*, 2000). Melatonin-mediated effects were attributed to the inhibiting and phase-shifting effect on SCN activity, but recently its effects on peripheral clock gene expression were shown ( Jung-Hynes *et al.*, 2010; Torres-Farfan *et al.*, 2006). Timed administration of melatonin might be beneficial to treat shift work-associated sleep disorders, but it is unknown how the circadian clock system is affected ( Carriedo-Diez *et al.*, 2022). One of the best studied hormones affecting tissue clocks are glucocorticoids. Glucocorticoid receptors are expressed in almost all tissues. While most tissue clocks can be reset by glucocorticoids, the SCN clock is unresponsive due to low receptor expression ( Rosenfeld *et al.*, 1988; Reddy *et al.*, 2007; Kiessling *et al.*, 2010; Balsalobre *et al.*, 2000). Glucocorticoids also regulate the expression of genes involved in glucose and lipid regulatory pathways in liver and adipose tissue, thereby controlling the maintenance of energy homeostasis ( Guia *et al.*, 2014). Time-restricted feeding has been shown to prevent some metabolic impairments under chronodisruptive conditions, but not all tissues are susceptible to food-related signals. Energy state-related hormones such as insulin, leptin, glucagon, ghrelin, and adiponectin are capable to impact clocks but mainly control energy homeostasis ( Martinez-Merlos *et al.*, 2004; Prosser and Bergeron, 2003; Sun *et al.*, 2015; Tahara *et al.*, 2011; Tsang *et al.*, 2020; Yannielli *et al.*, 2007). Rhythmic administration of adiponectin rescues diurnal variation in gene expression in the MBH and leads to body weight reduction ( Tsang *et al.*, 2020). These findings indicate that hormones may be useful to synchronize clocks and clock output in a tissue-specific manner which in turn ameliorates health outcomes. However, caution must be taken since most hormonal receptors are expressed in numerous tissues and, thus, it is impossible to target specific tissue clocks which in turn, may lead to undesirable side effects. Moreover, recent findings demonstrate that non-circadian signals alter tissue transcriptome rhythms without affecting the local clock. For example, elevated thyroid hormone levels result in dampened or abolished rhythms in glucose and lipid metabolism related genes in the liver but have no effect on clock gene expression ( de Assis *et al.*, 2022).

Furthermore, despite exploring the potential use of hormones to improve internal synchrony, the efficacy of synthetic clock modulators gained attention in recent years. One of such, a REV-ERB agonist, may be useful to target metabolic impairments but it will also affect clocks throughout the body and side effects are not yet examined ( Solt *et al.*, 2012). The ROR agonist nobiletin enhances circadian rhythm amplitude and improves energy homeostasis preventing metabolic syndrome in diet-challenged mice ( He *et al.*, 2016). There are many more small-molecule modulators of the circadian clock and the therapeutic efficacy of such is proven or under investigation (reviewed in Ribeiro *et al.*, 2021). To date, the clock machinery is assumed to be the same in all tissues, but there is evidence indicating that it is regulated in a tissue-specific manner ( Mure *et al.*, 2018). Global administration of small-molecule modulators of the circadian clock may have a differential effect on intra-tissue synchrony in which it will be beneficial for one tissue clock while worsening the phase relationship for another.

## Data Availability

No data are associated with this article.
