# Supplementary material for: Different levels of circadian (de)synchrony ­– where does it hurt?
Source: F1000Res. 2023 Apr 3;11:1323. Originally published 2022 Nov 15. [Version 2] doi: 10.12688/f1000research.127234.2 (PMC10130703; doi:10.12688/f1000research.127234.2)
Supplement: Peer review history

# Peer review: Reviewer response for version 2

**DOI:** 10.5256/f1000research.145648.r168565

**Published:** 2023-04-25

**Authors:** Frank A.J.L. Scheer

**Affiliations:** 1 Division of Sleep Medicine, Harvard Medical School, Harvard University, Cambridge, Massachusetts, USA; 2 Medical Chronobiology Program, Division of Sleep and Circadian Disorders, Departments of Medicine and Neurology, Brigham and Women's Hospital, Mass General Brigham Inc, Boston, Massachusetts, USA

The authors have addressed the feedback satisfactorily.

Is the review written in accessible language?

Yes

Are all factual statements correct and adequately supported by citations?

Yes

Are the conclusions drawn appropriate in the context of the current research literature?

Yes

Is the topic of the review discussed comprehensively in the context of the current literature?

Yes

Reviewer Expertise:

Chronobiology

I confirm that I have read this submission and believe that I have an appropriate level of expertise to confirm that it is of an acceptable scientific standard.

# Peer review: Reviewer response for version 2

**DOI:** 10.5256/f1000research.145648.r168566

**Published:** 2023-04-14

**Authors:** Andrew N. Coogan

**Affiliations:** 1 Department of Psychology, Maynooth University, Maynooth, Ireland

The authors have now made appropriate revisions in response to my comments, and I am pleased to endorse the revised manuscript.

Is the review written in accessible language?

Yes

Are all factual statements correct and adequately supported by citations?

Yes

Are the conclusions drawn appropriate in the context of the current research literature?

Yes

Is the topic of the review discussed comprehensively in the context of the current literature?

Yes

Reviewer Expertise:

Chronobiology

I confirm that I have read this submission and believe that I have an appropriate level of expertise to confirm that it is of an acceptable scientific standard.

# Peer review: Reviewer response for version 1

**DOI:** 10.5256/f1000research.139716.r161398

**Published:** 2023-02-23

**Authors:** Andrew N. Coogan

**Affiliations:** 1 Department of Psychology, Maynooth University, Maynooth, Ireland

This is a nicely written and timely review of the literature on circadian desynchrony, its mechanisms and its impacts on health. It provides a multi-level framework through which circadian desynchrony may be considered, and as such represents a very useful addition to the literature for researchers in chronobiology and adjacent areas.

 I have only minor comments and suggestions that could be considered for an update of the manuscript.

1. I would suggest adding a brief discussion of the very recently published studies on multi-tissue rhythms in humans (Wucher *et al.* (2023) ^1^ and Talamanca *et al.* (2023) ^2^). I appreciate that these papers have been published since the posting of the current version, but they are so pertinent to the concept of the review that an update might be warranted.
2. In general, whilst discussing the epidemiological evidence concerning shift work as a risk factor for various chronic diseases, I would note that the literature is more mixed than presented, and that findings of associations between shift work and various chronic health outcomes tend to be far from ubiquitous. I would also note that the exposure to shift work probably consists of elements other than circadian desynchrony/light-at-night exposure (such as shorter sleep duration, poorer sleep quality, increased alcohol and nicotine use as coping mechanisms, poorer diet quality). I think it would be helpful as such to contextualize the discussion of shift work and circadian desynchrony a little more.
3. I suggest that healthy physiological aging is a "condition" that may also warrant attention with reference to circadian desynchrony. There are a number of studies demonstrating that circadian desynchrony appears to be associated with "healthy" aging. Further, as many of the chronic conditions discussed in relation to desynchrony are age-related, it is important to consider circadian changes in the pre-morbid state. As such, when discussing evidence from disease models for desynchrony, I would find it useful if mention was made of the ages of the animals examined.
4. For Figure 2 and its associated discussion, I would find it useful if the " *misaligned zeitgebers*" were indicated as both misaligned with reference to each other (e.g. meal timing from light/dark cycles), and also misaligned with the internal circadian phases. I think this is implicit in the discussion, but suggest that it would be helpful to make these considerations explicit.
5. My preference would be for the consideration of social jetlag to be given more prominence, given its near ubiquitous nature at the population level as an everyday circadian desynchrony. I think it would also be helpful for the reader if indications of the non-trivial magnitude of social jetlag could be indicated in the manuscript. 

Is the review written in accessible language?

Yes

Are all factual statements correct and adequately supported by citations?

Yes

Are the conclusions drawn appropriate in the context of the current research literature?

Yes

Is the topic of the review discussed comprehensively in the context of the current literature?

Yes

Reviewer Expertise:

Chronobiology

I confirm that I have read this submission and believe that I have an appropriate level of expertise to confirm that it is of an acceptable scientific standard, however I have significant reservations, as outlined above.

## References

[1] : Day-night and seasonal variation of human gene expression across tissues. *PLoS Biol* .2023;21(2) : 10.1371/journal.pbio.3001986 e3001986 10.1371/journal.pbio.3001986 36745672PMC9934459

[2] : Sex-dimorphic and age-dependent organization of 24-hour gene expression rhythms in humans. *Science* .2023;379(6631) : 10.1126/science.add0846 478-483 10.1126/science.add0846 36730411

# Response

**Published:** 2023-03-15

**Authors:** Isabel Heyde

**Affiliations:** University of Lübeck, Germany

This is a nicely written and timely review of the literature on circadian desynchrony, its mechanisms and its impacts on health. It provides a multi-level framework through which circadian desynchrony may be considered, and as such represents a very useful addition to the literature for researchers in chronobiology and adjacent areas.

 I have only minor comments and suggestions that could be considered for an update of the manuscript.

**Comment:** I would suggest adding a brief discussion of the very recently published studies on multi-tissue rhythms in humans (Wucher *et al.* (2023) ^1^ and Talamanca *et al.* (2023) ^2^ ). I appreciate that these papers have been published since the posting of the current version, but they are so pertinent to the concept of the review that an update might be warranted.

**Response: We agree that these references serve as important recent additions to the field. We included these in the organization of the mammalian circadian clock system in the sections** **where we are highlighting the phase relationship between tissues and genes. However, we feel the need to point out the limitation of the study design while acknowledging the fact that the GTEx data set is so far the best data set available. The main limitation we find with this data set is that data were obtained from hospitalised patients which indirectly points out that there might be a high possibility of the patients being in a chronodisrupted health condition.**

**Comment**: In general, whilst discussing the epidemiological evidence concerning shift work as a risk factor for various chronic diseases, I would note that the literature is more mixed than presented, and that findings of associations between shift work and various chronic health outcomes tend to be far from ubiquitous. I would also note that the exposure to shift work probably consists of elements other than circadian desynchrony/light-at-night exposure (such as shorter sleep duration, poorer sleep quality, increased alcohol and nicotine use as coping mechanisms, poorer diet quality). I think it would be helpful as such to contextualize the discussion of shift work and circadian desynchrony a little more.

**Response: We added studies showing that shift work does not necessarily increase the risk for distinct diseases. Furthermore, we explained that the exposure to shift work does not only include circadian desynchrony/light-at-night exposure but also has a negative impact on the sleep quality and related coping mechanisms.**

**Comment:** I suggest that healthy physiological aging is a "condition" that may also warrant attention with reference to circadian desynchrony. There are a number of studies demonstrating that circadian desynchrony appears to be associated with "healthy" aging. Further, as many of the chronic conditions discussed in relation to desynchrony are age-related, it is important to consider circadian changes in the pre-morbid state. As such, when discussing evidence from disease models for desynchrony, I would find it useful if mention was made of the ages of the animals examined.

**Response: We thank the reviewer for** **this** **comment. We agree and briefly discuss the relationship between aging and circadian desynchrony and highlight the point that considering age of experimental animals should be given more importance. We do not explicitly mention the age of experimental subjects in every referred study. However, we do make a statement, before starting the main subject chapters, mentioning that all the cited studies were conducted in young adults of the respective model if not stated otherwise.**

**Comment:** For Figure 2 and its associated discussion, I would find it useful if the " *misaligned zeitgebers*" were indicated as both misaligned with reference to each other (e.g. meal timing from light/dark cycles), and also misaligned with the internal circadian phases. I think this is implicit in the discussion, but suggest that it would be helpful to make these considerations explicit.

**Response: We agree that this is an important point that was missed in the figure representation and have now corrected the figure by displaying different panels for both types of " *misaligned zeitgebers*”.**

**Comment:** My preference would be for the consideration of social jetlag to be given more prominence, given its near ubiquitous nature at the population level as an everyday circadian desynchrony. I think it would also be helpful for the reader if indications of the non-trivial magnitude of social jetlag could be indicated in the manuscript. 

**Response: Impact of social jetlag in everyday life was not given prominence in the original manuscript. We agree that this is a very valuable addition and now introduce the social jetlag concept in the introduction as well as discuss its effects at the population level in the following chapters.**

# Peer review: Reviewer response for version 1

**DOI:** 10.5256/f1000research.139716.r155847

**Published:** 2022-12-19

**Authors:** Frank A.J.L. Scheer

**Affiliations:** 1 Division of Sleep Medicine, Harvard Medical School, Harvard University, Cambridge, Massachusetts, USA; 2 Medical Chronobiology Program, Division of Sleep and Circadian Disorders, Departments of Medicine and Neurology, Brigham and Women's Hospital, Mass General Brigham Inc, Boston, Massachusetts, USA

***Editorial Note from F1000Research – 25th April 2023:** This peer review report was updated at the reviewer's request to add a Conflict of Interest statement in order to ensure full transparency.*

 This is a timely, comprehensive, and well-written review on circadian misalignment at different organismal levels and health consequences, with a primary review of animal experimental work supplemented with human work. The claims are well supported by references to original work. My comments are mostly minor.

- While this is done in many cases, it is important to specify the model and tissue. This is especially important because statements about timing of peaks and troughs may be opposite between nocturnal vs. diurnal mammals, and between SCN vs. extra-SCN oscillators. For example, statements such as " *circadian locomotor output cycles kaput (CLOCK) heterodimers bind to E-box promotor elements in period1–3 (Per1–3) and cryptochrome1/2 (Cry1/2) genes inducing their transcription in the morning*" need to indicate the model and tissue. Please do this throughout the manuscript.
- Figure 2, consider moving " *Inter-tissue desynchrony"* label to be with the third panel that is showing inter-tissue misalignment. Do you need the label “ *Intra-tissue desynchrony*”? This seems to refer to the same concept as “ *Cellular desynchrony*”, i.e., misalignment between cells in the same tissue. Consider using the same terminology throughout the manuscript, and the term "inter-cell" to clarify that it is distinct from the intra-cellular/molecular misalignment.
- For future directions, it will be helpful for the readers to lay out what type of studies and approaches will be most valuable to clarify the knowledge gaps. This is also true for the statement on page 9, “ *However, more studies investigating the effect of phase incoherences*… *are needed*…”.
- Introduction, first paragraph, replace " *period length*" with "period" and consider adding "(cycle length)" after "period".
- Introduction, second paragraph, do the authors mean "prevalence" instead of " *incidence*"?
- Introduction, second paragraph, “ *Possibly, this may be provoked by light-mediated resetting of the SCN*…”, clarify the rationale why SCN resetting, or melatonin suppression, would increase the risk for obesity or metabolic syndrome.
- Introduction, second paragraph, “ *Shift work is believed to affect*…”, the phase coherence doesn't need to be between two or more circadian rhythms but could also be between a circadian and a diurnal rhythm such as between the melatonin rhythm and the sleep/wake cycle or light/dark cycle.
- Page 4, “ *However, hepatocyte clocks in SCN ablated mice*…”, why would this show that peripheral tissue clocks are coupled? An alternative explanation could be that the feeding rhythm itself synchronizes hepatocytes, such that they are aligned even if they are not coupled among each other. Even though this alternative is recognized in the subsequent sentence, it will be helpful to acknowledge this in the first statement.
- Page 4, “ *Within each rhythmic cell, the molecular clock has a normal phase distribution*”, consider rephrasing that “…the components of the molecular clock have a phase distribution.”
- Page 6, “ *All of the above described real-life situations result in chronodisruption which promotes metabolic impairments but also increases the prevalence for major depression, cardiovascular disease, autoimmune disorders, and certain cancers*”, most of these cited studies are observational studies where other factors may also be involved, besides chronodisruption, that cause the increased disease risk, such as differences in socioeconomic status and lifestyle which will be important to acknowledge and that therefore experimental studies are needed to directly test cause-and-effect relationships with risk factors.
- Page 9, “ *Whereas all three genes react rapidly to LD delays*”, this sentence is incomplete.
- Page 9, bottom of the page, when discussing the effects of HFD, consider discussing whether the blunting of the feeding rhythm (which may be due to non-circadian factors, such as different dynamics of metabolism and saturation by fat vs. carbohydrates) may be the cause of the blunted clock gene rhythms, e.g., in adipose tissue and liver.
- Page 10, when discussing intra-cellular misalignment, please discuss to what degree the different speeds of re-entrainment following a shift between different clock genes may be due to specific clock genes acting as immediate early genes in response to the specific zeitgeber(s), which may also show transient changes in their levels in acute response to the zeitgeber.
- Page 10, as part of therapeutic approaches and future directions, you may want to briefly discuss timing of behavior as a therapeutic approach, e.g., food timing with promising experimental human studies including those addressing misalignment. You may also want to briefly mention the broader relevance for the timing of therapy/treatment, such as for surgery (Montaigne *et. al. *(2018) ^1^).
- Page 10, “ *It will be important to understand the mechanisms*…”, this paragraph describes approaches to follow cell-specific molecular rhythms under various conditions. Consider discussing experimental approaches to test whether misalignment between tissues contributes to negative health consequences.

Is the review written in accessible language?

Yes

Are all factual statements correct and adequately supported by citations?

Yes

Are the conclusions drawn appropriate in the context of the current research literature?

Yes

Is the topic of the review discussed comprehensively in the context of the current literature?

Yes

Reviewer Expertise:

Chronobiology

I confirm that I have read this submission and believe that I have an appropriate level of expertise to confirm that it is of an acceptable scientific standard, however I have significant reservations, as outlined above.

## References

[1] : Daytime variation of perioperative myocardial injury in cardiac surgery and its prevention by Rev-Erbα antagonism: a single-centre propensity-matched cohort study and a randomised study. *Lancet* .2018;391(10115) : 10.1016/S0140-6736(17)32132-3 59-69 10.1016/S0140-6736(17)32132-3 29107324

# Response

**Published:** 2023-03-15

**Authors:** Isabel Heyde

**Affiliations:** University of Lübeck, Germany

This is a timely, comprehensive, and well-written review on circadian misalignment at different organismal levels and health consequences, with a primary review of animal experimental work supplemented with human work. The claims are well supported by references to original work. My comments are mostly minor.

**Comment: **While this is done in many cases, it is important to specify the model and tissue. This is especially important because statements about timing of peaks and troughs may be opposite between nocturnal vs. diurnal mammals, and between SCN vs. extra-SCN oscillators. For example, statements such as " *circadian locomotor output cycles kaput (CLOCK) heterodimers bind to E-box promotor elements in period1–3 (Per1–3) and cryptochrome1/2 (Cry1/2) genes inducing their transcription in the morning*" need to indicate the model and tissue. Please do this throughout the manuscript.

**Response: We thank the reviewer for this comment and agree that experimental model and tissue type will largely influence circadian parameters of clock genes and have therefore indicated specific model and tissue where needed.**

**Comment: **Figure 2, consider moving " *Inter-tissue desynchrony"* label to be with the third panel that is showing inter-tissue misalignment. Do you need the label “ *Intra-tissue desynchrony*”? This seems to refer to the same concept as “ *Cellular desynchrony*”, i.e., misalignment between cells in the same tissue. Consider using the same terminology throughout the manuscript, and the term "inter-cell" to clarify that it is distinct from the intra-cellular/molecular misalignment.

**Response: We have relabelled " *Inter-tissue desynchrony"* for third panel in Figure 2 as suggested. With regard to “ *Intra-tissue desynchrony*”, we prefer to keep the label as well as the corresponding reference in the text since we want to emphasize that inter-cell and molecular desynchrony both can be grouped under this umbrella term.  We changed “ *Cellular desynchrony*” to "Inter-cell desynchrony" to avoid confusion.**

**Comment:** For future directions, it will be helpful for the readers to lay out what type of studies and approaches will be most valuable to clarify the knowledge gaps. This is also true for the statement on page 9, “ *However, more studies investigating the effect of phase incoherences*… *are needed*…”.

**Response: We now elaborate on these in the last paragraph of the inter-tissue synchrony chapter and provide more detailed ideas for future directions. Please also see our response to the last comment for more details.**

**Comment:** Introduction, first paragraph, replace " *period length*" with "period" and consider adding "(cycle length)" after "period". Introduction, second paragraph, do the authors mean "prevalence" instead of " *incidence*"?

**Response: We thank the reviewer for these comments and changed the text accordingly. We changed the phrase from 'period length' to 'period (cycle length)' in the text.** **The cited studies investigated the associations between shift work/circadian disruption and the health outcome and, thereby, explored the 'incidence' so we replaced the word 'prevalence'.**

**Comment:** Introduction, second paragraph, “ *Possibly, this may be provoked by light-mediated resetting of the SCN*…”, clarify the rationale why SCN resetting, or melatonin suppression, would increase the risk for obesity or metabolic syndrome.

**Response: We added some more information. Briefly, LAN dampens clock gene rhythms in the SCN and may shift food intake patterns. Furthermore, we highlighted the role of melatonin in insulin biosynthesis and the regulation of leptin rhythms.**

**Comment:** Introduction, second paragraph, “ *Shift work is believed to affect*…”, the phase coherence doesn't need to be between two or more circadian rhythms but could also be between a circadian and a diurnal rhythm such as between the melatonin rhythm and the sleep/wake cycle or light/dark cycle.

**Response: We thank the reviewer for this thoughtful comment. Indeed, phase coherence can be disturbed between two or more circadian rhythms but, as pointed out, also between circadian and diurnal rhythms. We added this in the revised manuscript.**

**Comment: **Page 4, “ *However, hepatocyte clocks in SCN ablated mice*…”, why would this show that peripheral tissue clocks are coupled? An alternative explanation could be that the feeding rhythm itself synchronizes hepatocytes, such that they are aligned even if they are not coupled among each other. Even though this alternative is recognized in the subsequent sentence, it will be helpful to acknowledge this in the first statement.

**Response: We completely agree that the aligned circadian oscillations in hepatocytes do not necessarily imply the presence of a coupling mechanism. The feeding rhythm plays a major role. Therefore, we have rephrased the text to make this clear. However, the study of Guenther *et al.* suggests that there is a weak coupling in peripheral oscillators so it cannot be ruled out that coupling contributes to the results observed by Saini *et al.*.**

****

**Comment: **Page 4, “ *Within each rhythmic cell, the molecular clock has a normal phase distribution*”, consider rephrasing that “…the components of the molecular clock have a phase distribution.”

**Response: We thank the reviewer for this **valuable comment. We rephrased this sentence.** 

**Comment:** Page 6, “ *All of the above described real-life situations result in chronodisruption which promotes metabolic impairments but also increases the prevalence for major depression, cardiovascular disease, autoimmune disorders, and certain cancers*”, most of these cited studies are observational studies where other factors may also be involved, besides chronodisruption, that cause the increased disease risk, such as differences in socioeconomic status and lifestyle which will be important to acknowledge and that therefore experimental studies are needed to directly test cause-and-effect relationships with risk factors.

**Response: We agree that many other factors may promote the development of the listed diseases which were not mentioned in the original manuscript. Therefore, we included them in a brief explanation.**

****

**Comment:** Page 9, “ *Whereas all three genes react rapidly to LD delays*”, this sentence is incomplete.

**Response: Thank you for pointing this out. We corrected this in the revised version.**

**Comment:** Page 9, bottom of the page, when discussing the effects of HFD, consider discussing whether the blunting of the feeding rhythm (which may be due to non-circadian factors, such as different dynamics of metabolism and saturation by fat vs. carbohydrates) may be the cause of the blunted clock gene rhythms, e.g., in adipose tissue and liver.

**Response: We agree that dampening of clock genes may be an effect of overall dampening of feeding rhythms and have included this speculation as a brief statement.**

****

**Comment: **Page 10, when discussing intra-cellular misalignment, please discuss to what degree the different speeds of re-entrainment following a shift between different clock genes may be due to specific clock genes acting as immediate early genes in response to the specific zeitgeber(s), which may also show transient changes in their levels in acute response to the zeitgeber.

**Response: We briefly discuss the re-entrainment speed of particular clock genes upon photic stimulation in the SCN and point out that this may partly explain the different speeds in re-entrainment seen in distinct tissues.**

**Comment: **Page 10, as part of therapeutic approaches and future directions, you may want to briefly discuss timing of behavior as a therapeutic approach, e.g., food timing with promising experimental human studies including those addressing misalignment. You may also want to briefly mention the broader relevance for the timing of therapy/treatment, such as for surgery (Montaigne *et. al. *(2018) ^1^ ).

**Response: We agree that time-restricted eating is an important tool to modulate the clock and improve metabolism and, therefore, added some information on the potential of time-of-eating as a therapeutic approach.**

**Comment:** Page 10, “ *It will be important to understand the mechanisms*…”, this paragraph describes approaches to follow cell-specific molecular rhythms under various conditions. Consider discussing experimental approaches to test whether misalignment between tissues contributes to negative health consequences.

**Response: We discuss the use of chimeric mice inheriting slow and normal (24-hour) clocks in distinct tissues. Furthermore, it is not known yet whether tissue-specific clock KO mice suffer from inter-tissue desynchrony which may promote/evoke the observed phenotypes. Established forced desynchrony paradigms may be useful to study the effects of inter-tissue desynchrony on health in mice. Unfortunately, in humans we so far are lacking good biomarkers for peripheral tissue clocks. We point out the need for those in the paragraph and the necessity to study desynchrony in different genders, ages, ethnicities and lifestyles to evaluate the effect on health.**
